# Classification of Brain Tumor Images Using CNN

**DOI:** 10.1155/2023/2002855

**Published:** 2023-10-12

**Authors:** Manali Gupta, Sanjay Kumar Sharma, G. C. Sampada

**Affiliations:** ^1^Department of Computer Science, SOICT, Gautam Buddha University, Greater Noida 201312, India; ^2^Cedargate Technologies, Kathmandu, Nepal

## Abstract

A brain tumor is a serious malignant condition caused by unregulated as well as aberrant cell partitioning. Recent advances in deep learning have aided the healthcare business, particularly, diagnostic imaging for the diagnosis of numerous disorders. The most frequent and widely utilized machine learning model for image recognition is probably task CNN. Similarly, in our study, we categorize brain MRI scanning images using CNN and data augmentation and image processing techniques. We compared the performance of the scratch CNN model with that of pretrained VGG-16 models using transfer learning. Even though the investigation is carried out on a small dataset, the results indicate that our model's accuracy is quite successful and has extremely low complexity rates, achieving 100 percent accuracy compared to 96 percent accuracy for VGG-16. Compared to existing pretrained methods, our model uses much less processing resources and produces substantially greater accuracy.

## 1. Introduction

The human body is composed of several types of cells. Each cell has a specific role. To produce new cells, the body's cells develop and divide methodically. These new cells contribute to the health and function of the human body. When cells lose the capacity to regulate their own growth, they expand in an uncontrolled manner. A tumor is the mass of extracellular tissue that forms a tumor. Tumors might be benign or malignant. Malignant tumors cause cancer, whereas benign tumors are not carcinogenic. Medical image data acquired from diverse biomedical devices employing different imaging modalities are essential diagnostic factors. Magnetic resonance imaging (MRI) is a technology that detects magnetic flux vectors and radiofrequency pulses in the nuclei of hydrogen atoms in the water molecules of a patient's body. Diagnostically, the MRI scan is better than the CT scan since it does not employ radiation. The MRI may be used by radiologists to assess the brain. The MRI is capable of detecting the existence of brain tumors [1, 2]. In addition, operator engagement in the MRI may result in inaccurate categorization due to noise. Due to the vast amount of MRI data that must be analyzed, less costly automated approaches are necessary. A great degree of precision is essential when dealing with human life, which is why the automatic diagnosis of malignancies on MR images is crucial. Using supervised and unsupervised machine learning algorithm techniques, it is possible to classify MR pictures of the brain as normal or pathological. Using machine learning methods, this work provides an effective automated categorization approach for brain MRI data. The supervised machine learning approach is used to classify MR images of the brain [3, 4].

## 2. Literature Review

Avşar and Salcin [5] proposed an approach for identifying early-stage brain tumors. MRI images were analyzed to detect tumor-containing regions and classify these regions according to a variety of tumor types. For picture categorization, deep learning yields rather excellent results. As a consequence, the CNN technique was used and implemented utilizing the TensorFlow framework in this study. It has been proved that the faster CNN method can obtain a 91.66 percent accuracy rate, which is higher than earlier research.

Sarkar et al. [6] proposed using MRI scans to identify the kind of brain tumor. Meningioma, glioma, and pituitary tumors were identified with an accuracy rate of 91.3% using a 2D CNN for classification. The study dataset included information on the three most prevalent forms of brain tumors.

Ranjbarzadeh et al. [7] suggested a flexible and effective approach for segmenting brain tumors. This technique decreases processing time while resolving overfitting difficulties in a cascading deep learning model. Using two independent routes, this CNN model captures both locally and globally relevant properties. In addition, the accuracy of tumor segmentation was greatly improved in comparison to current models. Our proposed method produces average WT, growing tumor, and total tumor core dice values of 0.9203, 0.9113, and 0.8726, respectively.

Kokila et al. [8] created a model to detect brain tumors using MRI. It involves locating the tumor, establishing its grade and kind, and pinpointing its position. Instead of using a distinct model for each classification task, our technique organized brain MRI data for many classification tasks using a single model. CNN is capable of categorizing and detecting tumors; hence, the multitask classification relies on CNN's classification and detection abilities. A CNN-based algorithm may also be used to determine the location of brain tumors with an accuracy of 92 percent.

The regularized extreme learning machine was utilized by Gumaei et al. [9] to create the brain tumor division (RELM). Photos were first preprocessed so that the algorithm could comprehend them simply. For preprocessing, the system adopted the min-max approach. This min-max preprocessing technique was highly effective in boosting the brightness of input images.

Kaplan et al. [10] used both methods to classify and detect brain tumors. The first suggested method was the local binary pattern (LBP) centered on the neighborhood distance relation known as nLBP, as well as the second method known as LBP centered on the angles between neighbors. These two techniques were used to preprocess MRIs of the three most prevalent forms of brain tumors: glioma, meningioma, and pituitary tumor. For character development, preprocessed picture statistics were used. This revised model outperformed conventional feature extraction strategies.

Pallavi et al. [11] used an automated method of deep CNN modelling, which is made up of six learnable layers and helps in automating feature learning from MRI images of the brain. This method involves little preprocessing and does not employ handmade features, and the proposed method may be applied for various MRI classifications.

## 3. Proposed Methodology

**Figure 1:**
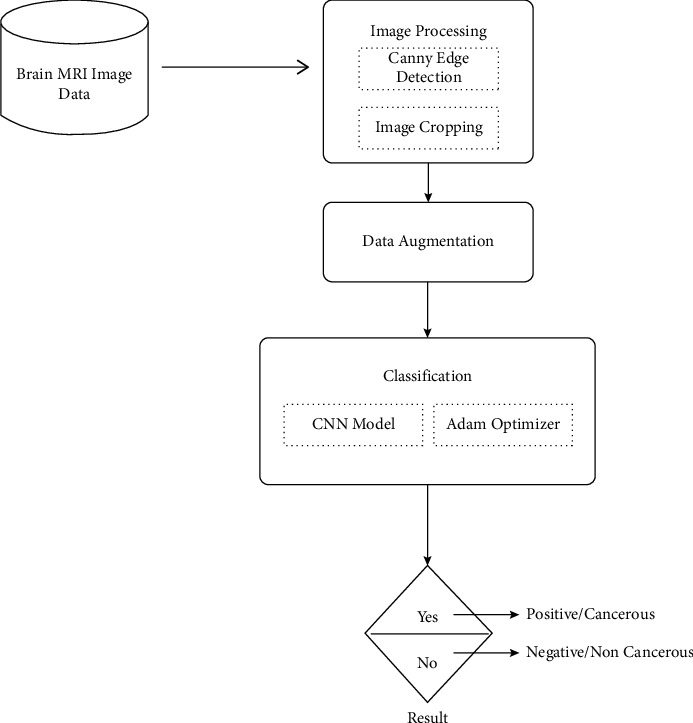
Illustration of the suggested technique [12].

Throughout this work, 253 MRI images were processed using image processing algorithms [12]. We trained neurons using a basic 8-layer CNN model and compared the results of our CNN model built from scratch with those of a VGG-16 machine utilizing transfer learning. The collection contains 155 images of malignant tumors and 98 shots of benign, noncancerous tumors. Our data were categorized into three groups: learning, verification, and evaluation. The machine learning technique may be used to train models, whereas the test dataset is often used to analyze models and alter parameters. Finally, the test results would be utilized to assess the models. Our proposed technique is broken into many steps.  Figure 1 shows an overview of the proposed approach [12].

### 3.1. Image Processing

**Figure 2:**
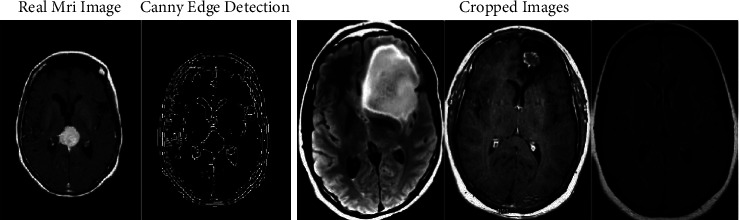
Detecting edges utilizing edge detection as well as cropping the brain section [13].

We used an open-source software computer vision- (CV-) based Canny edge detection [13] technique to extract just the brain area from MRI data. This would be a multiphase approach for detecting the edges of objects in photographs.  Figure 2 displays the True MRI brain boundaries as determined by the aforementioned method, with just the brain portion of the pictures cropped [13].

### 3.2. Data Enhancement

**Figure 3:**
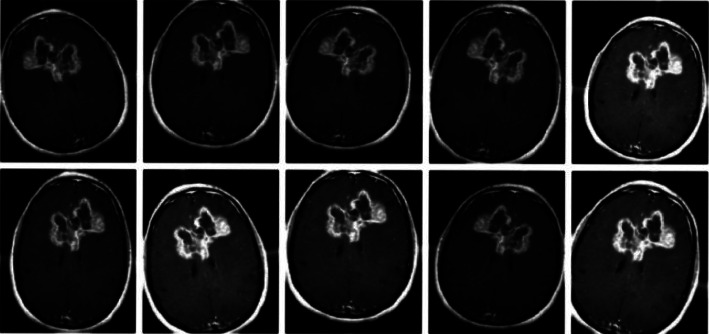
Data enhancement [15].

Data augmentation would be a method of intentionally boosting the volume and variety of known information [14]. We understand that fine-tuning the parameters of a deep learning model requires a vast quantity of data. However, since our dataset remains tiny, we used the data preprocessing approach [15] upon our training sample, introducing small adjustments to our images including flips, rotations, and intensity. This would improve the volume of our dataset, and our model would treat every one of these little changes as a separate image, allowing the model to learn and function well.  Figure 3 shows a collection of augmented images derived from a single image [15].

### 3.3. CNN Model

**Figure 4:**
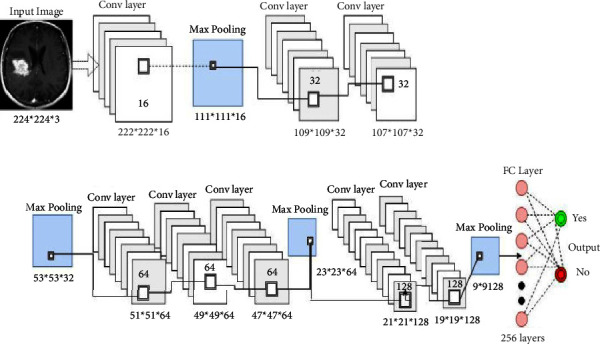
Proposed CNN model [16].

In this work, we suggested a basic CNN model and used it to retrieve enhanced MRI data images with an RGB color channel and a batch size comprising 32. We started as a single 16-filter convolution layers with a filter size = 3333. The purpose for using just 16 filters is to identify corners, edges, as well as lines. Next, we inserted a max-pooling level with a 2222 filtering to extract the maximum summation of that image; subsequently, we expanded the convolution layers and filters up to 32, 64, and subsequently 128, with almost the same size of filter, i.e., 3333. As even the quantity of filters rises, this merges these little patterns to create larger patterns such as circles, squares, and so on. To make best of the situation, we added max-pooling layers of convolution layer. Furthermore, we used a completely integrated dense layer of 256 neurons in conjunction with a softmax output level to compute the probability estimate for every class and classify the ultimate decision labeling as Yes/No based on whether the incoming MRI image includes cancer or just does not contain tumor. The configuration of our suggested CNN architecture can be seen in Figure 4 [16].

### 3.4. Transfer Learning

**Figure 5:**
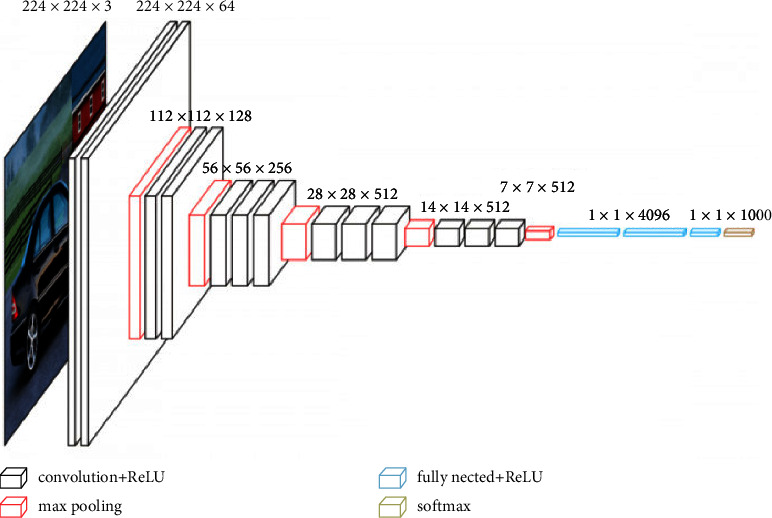
Architecture of VGG-16 model [17].

Rather than constructing a new CNN model, deep learning uses a pretrained CNN classification algorithm that has already been trained on a big dataset such as “ImageNet.” Pan and Yang [16] presented a paradigm for gaining a better understanding of learning algorithms. Transfer learning takes advantage of past information rather than starting the learning program from new. Considering our dataset was so small, we utilized a pretrained VGG-16 CNN model that was perfectly alright by freezing parts of the layers to minimize the fitting problem. Karen Simonyan as well as Andrew Zisserman introduced the VGG-16 CNN architecture, comprising 16 convolutional layers, during 2014 [17]. This same input geometry of the network image would be as input. All across the network, it has 16 convolution layers and a filter size of equivalent to 3333 and also having 5 pooling layers with a size equal to 2222. Two entirely connected layers with just a softmax output level sit on top. With almost 138 million variables, the VGG-16 model would be a massive network. It builds deep neural networks by layering several convolutional layers, which improves the capacity for learning hidden characteristics.  Figure 5 depicts the VGG-16 network design [17].

## 4. Results and Discussion

We conducted experiments on the brain tumor MRI images database published by Chakrabarty [12]. The collection, which is open to the public, comprises of 253 genuine brain scans created by radiologists employing data from actual patients. It is accessible on Kaggle, a platform hosting machine learning contests that shares data. The data are separated among learning, verification, and test datasets. There are 185 photos for learning, 48 for verification, and 20 for evaluation to prove the correctness of our model. Data augmentation is also employed to enhance our dataset by making tiny modifications to existing MRI pictures, which are subsequently retrieved using our proposed CNN model. We trained these models in 32-person groups over 15 iterations. Python's TensorFlow and Keras packages are used to run the test on a Core i5 CPU with 8 GB RAM running at 2.3 GHz. Our suggested model was 96 percent accurate with training data and 89 percent accurate with test data. To put our CNN model to the test, we employed transfer learning to train previously taught VGG-16 CNN models on the same dataset [18]. VGG-16 obtained a 90% accuracy on training data and an 87.5% accuracy on testing data.

We put our model to the test using a previously unknown training dataset. False positive and false negative indicate incorrect classification, with FP indicating normal brain images are positive tumors and FN indicating abnormal brain images are negative tumors. The results are shown in Table 1.

## 5. Conclusion

A unique categorization strategy for brain tumors is being offered in this work. First, we detected the ROI in MRI images using image edge detection and then cropped them [19]. Following that, we used the data preparation procedure to expand the size of our datasets. Second, we propose a basic CNN network as an effective classification approach for brain tumors. Neural network training needs a significant amount of information for complex and accurate outputs; however, our experimental outcomes demonstrate that we can achieve 100% accuracy even with a very tiny dataset, as our accuracy rate is higher than that of VGG-16. Our suggested approach may aid in the prognostic relevance of tumor identification in individuals with brain malignancies [20]. As we have used a large amount of information, it took more time to calculate the results in comparison to traditional brain tumor classification models which we will handle in future work.

## Figures and Tables

**Table 1:** Model classification performance.

Model used	True negative	True positive	False negative	False positive	Precision	Accuracy	Recall	Time (sec)
CNN proposed	14	14	0	0	1.0	1.0	1.0	3085
VGG-16	13	14	0	1	0.93	0.96	1.0	6846

## Data Availability

Dataset is publicly available on UCI Repository.
